# Usefulness of lymphocyte transformation test and in vitro cytokine release in differentiating between independent and cross‐reacting nickel/palladium allergy

**DOI:** 10.1002/iid3.329

**Published:** 2020-07-28

**Authors:** Florian Kapp, Burkhard Summer, Peter Thomas

**Affiliations:** ^1^ Department of Dermatology and Allergy Ludwig Maximilians University Munich Germany

**Keywords:** cross‐reactivity, IL‐5, LTT, metals, nickel, palladium

## Abstract

**Background:**

Often concomitant patch test (PT) reactivity to palladium (Pd) and nickel (Ni) is found.

**Objectives:**

To determine whether lymphocyte transformation test (LTT) could be useful in discrimination between cross‐reacting or distinct PT results, and to compare the results with in vitro cytokine production upon Pd or Ni stimulation.

**Materials and Methods:**

The study population consisted of two groups: 13 individuals with Pd PT reactions (10 with concomitant Ni PT reaction, 3 individuals with only Pd PT reactivity) and 10 Ni/Pd PT negative individuals. LTT and assessment of cytokine release (interferon‐gamma, interleukin‐5 [IL‐5], IL‐8, IL‐17A, tumor necrosis factor alpha) by cytometric bead assay were performed.

**Results:**

All 10 patients with positive PT to Ni and Pd showed positive LTT to Ni (*P* < .05) as compared with the 10 Pd/Ni PT negative patients—but had no significant LTT reaction to Pd. In all, 9 out of 10 Pd/Ni PT negative patients were also LTT negative to Ni and 10 out of 10 to Pd. In the 3 only Pd PT reactors 2 out of 3 remained LTT negative to Ni and 0 out of 3 to Pd. As a major finding, cytokine production gave clearly enhanced IL‐5 response to Ni in Ni PT positive individuals (*P* < .05), whereas Pd PT reactivity was not linked with such enhanced IL‐5 production in vitro to Pd.

**Conclusions:**

Pd and Ni sensitization are mostly found concomitantly, and cross‐reactivity is questioned. By different LTT reactions and particularly IL‐5 production in vitro, predominant Ni sensitization becomes more evident.

## Graphical abstract

Pd and Ni sensitization are mostly found concomitantly. By different interleukin‐5 production in vitro, predominant Ni sensitization becomes more evident.

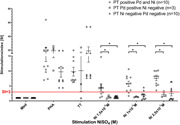

## 1 INTRODUCTION

The main exposure of the general population to palladium (Pd) is by jewelry and dental applications. About 8% of the growing annual Pd production is used for dental materials alone.^1^, ^2^ As Pd alloys undergo corrosion, released metal ions may provoke contact allergic reactions both in occupational and private setting.^3^ Correspondingly, a recent data evaluation of the information network of departments of dermatology (IVDK) reported Pd(II) chloride as most frequent (ie, 3%) allergen of the “dental metal series” detected in dental technicians with occupational contact dermatitis.^4^ In the oral cavity, saliva may facilitate corrosion of typically Pd containing dental materials like dental crowns and bridges. Experience of a Norwegian ambulatory for dental material intolerance gives high allergy rates to nickel (Ni) (28%) and gold (Au) (23%), but also to Pd (9%).^5^ Muris et al^6^ reported that patients with Pd‐based dental materials more often presented with Pd reactivity in patch testing and lymphocyte reactivity in vitro. Several authors found a strong relationship between Ni and Pd allergy, and cross‐reactivity of sensitized T cells was suspected.^7^, ^8^, ^9^, ^10^ Early experiments by Pistoor et al^9^ show such cross‐reactivity of Ni‐reactive T‐lymphocyte clones. Hindsen et al^11^ reported the in vivo correlate of such cross‐reactivity by systemic Ni administration. Both authors discuss that primary sensitization was to Ni. In addition, Ni and Pd have another property in common as they are described to induce T‐cell activation also by direct binding to TLR4, according to Rachmawati et al and Schmidt et al^12^, ^13^ and Pd allergy has been a subject of debate for decades and its prevalence is supposed to be underestimated.^14^, ^15^, ^16^ Moreover, Muris et al^6^ reported that by use of a different patch test (PT) preparation, that is, Na_2_PdCl_4_, there was a substantial gain in positive reactions. In fact, the “gold” standard method to confirm the clinical suspicion of metal allergy is the PT. In particular for Ni allergy, the lymphocyte transformation test (LTT) was described by various authors as a valid tool to prove Ni sensitization in vitro.^17^, ^18^, ^19^, ^20^, ^21^, ^22^, ^23^ In addition, some authors have optimized Ni LTT conditions to improve validity as compared with PT results.^19^, ^24^

Analysis of cytokine production by Ni‐specific T cells has suggested a mixed Th1 and Th2 cytokine production in both T‐cell clones and peripheral blood mononuclear cells (PBMCs).^17^, ^20^, ^21^, ^23^ It was speculated that the type of cytokine response to stimulation could modulate the resulting immune response.^25^, ^26^ Therefore, the allergen‐induced cytokine production such as interferon‐gamma (IFNγ), interleukin‐2 (IL‐2), IL‐12 (TH1‐related), IL‐4, IL‐5, IL‐13 (TH2‐related), and TH17 associated IL‐17A as well as tumor necrosis factor alpha (TNF‐α) and IL‐8 was measured in supernatants of stimulated PBMC.^20^, ^27^, ^28^, ^29^, ^30^

To our knowledge, results of LTT and PT with Ni and Pd have rarely been directly compared. Thus, the aim of our study was (a) to evaluate a potential link between Ni/Pd reactivity in PT and LTT and (b) to determine whether in vitro cytokine response to Ni and Pd might further distinguish cross‐reacting from isolated single Pd sensitization.

## 2 MATERIALS AND METHODS

### 2.1 Patients

Within the total number of patients patch tested every year in the allergy unit of the Munich dermatology clinic, there is always a series of patients being tested to standard and dental metal series (series DKG17), including Pd(II) chloride. Between March 2016 and March 2017, there were 70 of such patients (12 males [m], 58 females [f], mean age 61 ± 12.7 years) who underwent patch testing to dental metal series. Based on positive or negative PT reaction to Pd, 23 individuals (4 m, 19 f, mean age 63 ± 9.5 years) within this consecutive patient series were recruited to study the in vitro reactivity to Pd. All 23 patients gave their written consent to blood donation for this investigation. The study was approved by the local ethics committee. The 23 individuals consisted of 13 patients with Pd allergy (10 out of 13 with additional Ni allergy, 3 out of 13 isolated Pd allergy); 10 individuals without Pd or Ni allergy. All of them had Pd containing dental materials such as crowns, bridges, or inlays (as communicated to them by their treating dentist), and one patient had Pd containing wedding ring as an elicitor of local eczema.

From all 23 individuals, questionnaire‐based allergy history was obtained. This included atopic diseases (allergic rhinoconjunctivitis, allergic asthma, atopic eczema); cutaneous metal intolerance reactions (such as eczema caused by jewelry, wrist watches); oral complications in association with the dental bridges, crowns, and dental alloys. The data are summarized in Table 1.

**Table 1:** Characteristics of the 23 patients included for in vitro experiments (13 patients with Pd PT reaction; 10 controls, Pd and Ni PT negative)

	Patients with positive Pd PT^a^	Control patients
Age	41‐75 y, mean 62.3 y	45‐76 y, mean 64.8 y
Sex	12 f, 1 m	7 f, 3 m
Reason for patch testing	12/13 DMI, 1/13 WRI	10/10 DMI
History of suspected Ni allergy^b^	13/13	0/10
Atopic diseases^c^	6/13	2/10

Abbreviations: DMI, dental material intolerance; f, females; m, males; PT, patch test; WRI, wedding ring intolerance (ie, eczema to Pd‐based wedding ring).

a In all, 10 out of 13 with additional Ni PT reaction.

b History of itching, erythema, eczema upon contact with jewelry, wrist watch, and jeans button.

c Allergic rhinoconjunctivitis, allergic asthma and/or atopic eczema.

### 2.2 Patch test

Patch testing was performed according to the guidelines of the German Contact Dermatitis Research Group (DKG) on the patient's upper back.^31^ Application time was 2 days, and reading was done accordingly after 2, 3, and 6 days. The cutaneous reactions were documented as negative (no reaction), or positive by grading +, ++, and +++. Doubtful and irritant reactions were also recorded but considered as negative PT result. Test series were the German standard series, a supplemental series, and at least the dental metal series containing the Pd(II) chloride (1%) preparation. This means the official “palladium test reagent” given by the DKG was the Pd(II) chloride preparation. Some patients were additionally tested to the “dental technicians series” containing acrylate and additive preparations. Most of the PT preparations were from SmartPractice Europe (Reinbek, Germany). According to the PT test results to Pd within the 70 patients (see Table 2), the final 13 Pd PT positive patients for the study had been subsequently recruited.

**Table 2:** Patch test reactivity of the 70 consecutive patients tested to at least standard series and dental metals series

		Pd(II) chloride		Total
		Negative	Positive	
Nickel(II) sulfate	Negative	52	3	55
	Positive	4	11	15
Total		56	14	70

*Note*: In all, 14 had reacted to Pd(II) chloride, 15 to Ni(II) sulfate, and 11 out of 14 Pd reactors also had Ni allergy.

### 2.3 Cell preparation and LTT

PBMCs were isolated from heparinized venous blood samples (40 mL) by Ficoll‐Hypaque (Phadia, Freiburg, Germany) density gradient centrifugation. The cells were resuspended at 1 × 10^6^/mL in 10% human autologous serum‐containing RPMI 1640 medium supplement with HEPES (25 mM), l‐glutamine, antibiotic‐antimycotic solution (Gibco International, Karlsruhe, Germany), NEAA and MEM vitamins (Biochrom, Berlin, Germany). The LTT was performed according to Summer et al.^20^, ^21^ Cultures were prepared in 96‐well round‐bottom polystyrene culture plates (Nalge Nunc International, Denmark) in parallel to assess proliferative response (“radioactive assay”) and cytokine production (“nonradioactive assay”). Cells were cultivated in quadruplicate for 6 days. The following stimuli were used: As controls T‐cell mitogen phytohaemagglutinine (PHA, 2.4 µg/mL; Biochrom) and tetanus toxoid as control recall antigen (TT, 5 µg/mL; Chiron Behring, Berlin, Germany); PdCl_2_ (Sigma‐Aldrich, Darmstadt, Germany)—to cover the best stimulatory range—in the following nine concentrations: 1.0 mmol, 5.0 × 10^−1^ mmol, 2.5 × 10^−1^ mmol, 1.25 × 10^−1^ mmol, 6.25 × 10^−1^ mmol, 3.13 × 10^−2^ mmol, 1.56 × 10^−2^ mmol, 7.81 × 10^−3^ mmol, and 1.91 × 10^−4^ mmol. NiSO_4_ was used in the following previously already evaluated concentrations^19^: 2.5 × 10^−2^mmol, 1 × 10^−2^mmol, 7.5 × 10^−3^mmol, and culture medium alone as additional control. On day 5, cells were pulsed with ^3^H thymidine overnight, proliferation was assessed on day 6 by incorporated radioactivity. The proliferative response was expressed as a stimulation index (SI), which was calculated by ratio of mean counts per minute (cpm) of stimulated compared with unstimulated cultures.^20^ SI > 3 was regarded as positive. In the parallel “nonradioactive” cultures, supernatants were collected at day 6 after identical stimulation. The one exception was using instead of PHA, a mixture of phorbol‐myristate‐acetate (PMA 15 ng/mL; Sigma‐Aldrich, Munich, Germany) and ionomycin (1.5 µg/mL; Sigma‐Aldrich) as a positive control. The nonradioactive cultures were also performed in quadruplicate.

### 2.4 Analysis of cytokine production

The supernatants of quadruplicate experiments were pooled and tested for the following proinflammatory cytokines: IFNγ (TH1 profile), IL‐5 (TH2), IL‐17A (TH17), and IL‐8 (unspecific immune response). Cytokine production was determined by use of fluorescent antibody marked microparticles in a cytometric bead assay (BD Biosciences, Heidelberg, Germany) and flow cytometry according to the manufacturer's instructions (detection limit >0.5 pg/mL). Cytokine levels upon stimulation were set in relation to baseline cytokine production in unstimulated cultures (medium only) as previously published.^32^

### 2.5 Statistical analysis

Statistical analysis was done using IBM SPSS software (IBM, Ehningen, Germany). The analysis was done using the Mann‐Whitney *U* test with a significance level of *P* < .05.

## 3 RESULTS

### 3.1 PT reactivity

Within the initial consecutive 70 patients, 15 had PT reactivity to Ni, 14 to Pd, and 11 out of 14 had concomitant Ni reactivity. The data with regard to Ni and Pd reactivity are summarized in Table 2. Out of these 14 Pd reactors, 13 consented to blood donation for in vitro experiments. Their Pd reactivity was one patient +++, five ++, and seven +. Three patients were monosensitized to Pd, 10 had concomitant Ni reactivity. The 10 “controls” were patients without PT reactivity both to Ni and Pd. Thus we divided the total of 23 patients into three groups: group 1 = 10 patients with Pd and Ni PT reactivity; group 2 = 3 patients with only Pd PT reactivity; group 3 = 10 patients with no PT reactivity. The characteristics of these 23 patients are shown in Table 3.

**Table 3:** Characteristics of the three patient groups who took part in the in vitro experiments

Age, sex	Type of Pd containing material	Clinical symptoms	PT		Allergy history
			Pd	Ni	
Group 1					
63, f	Crown/bridges	Pain, burning, redness	+	++	AR
75, m	Dental implant, crown/bridges	Pain gingivitis	+	++	None
72, f	Dental implant	Pain, redness, gingivitis	++	+	AE
71, f	Dental prosthesis	Pain, burning, erythema, gingivitis, gingiva swelling	+	++	None
69, f	Dental implant, crown/bridges	Sensation of heat, pain, swelling	+	++	AR
65, f	Dental implant	Itching, erythema	++	+++	None
57, f	Crown/bridges	Pain, erythema, gingivitis	+	++	None
58, f	Dental implant, crown/bridges	Aphthae	+++	+	None
68, f	Crown, bridges	Gingivitis	++	+++	AE
57, f	Dental implant, crown/bridges	Pain, burning, redness, gingivitis	++	+++	None
Group 2					
52, f	Dental implant, crown/bridges	Pain, gingivitis	+	−	AE, AR, AA
63, f	Dental implant, crown/bridges	Pain, redness, gingivitis	+	−	AR
41,f	Wedding ring	Eczema	++	−	None
Group 3					
64, f	Dental implants	Gingivitis	−	−	None
68, m	Dental implant, crown/bridges	Pain, gingivitis	−	−	None
67,f	Dental prosthesis	Burning	−	−	None
58, m	Crown/bridges	Pain, burning	−	−	AR
61, f	Dental prosthesis	Pain, gingivitis	−	−	None
61, f	Dental implants	Gingivitis	−	−	None
75, m	Dental implant, crown/bridges	Pain, gingivitis	−	−	None
76, f	Dental prosthesis	Burning	−	−	None
73, f	Dental prosthesis	Burning, erythema	−	−	None
45, f	Dental implant, crown/bridges	Pain, burning erythema	−	−	AR

Abbreviations: AA,  allergic asthma; AE, atopic eczema, AR,  allergic rhinitis; f, females; m, males.

### 3.2 Lymphocyte proliferative response (LTT reactivity)

The LTT reactivity to Pd and to Ni was evaluated in relation to the “gold standard” of respective positive PT. Since frequently concomitant PT reactions to Pd and Ni were found, we intended to detect potential different LTT proliferative reaction patterns by using two approaches, that is, (a) stimulation with PdCl_2_ and NiSO_4_ and (b) comparing the three groups. When using PBMC of patients and controls, the control stimuli showed a significant mean proliferation response (SI) to PHA (group 1: 23.25 ± 6.9; group 2: 19.11 ± 4.67; group 3: 10.52 ± 2.87) and the SI to TT reflected the varying immunization status of the patients (group 1: 9.55 ± 1.68; group 2: 10.11 ± 7.01; group 3: 17.65 ± 7.23.)

**Figure 1:**
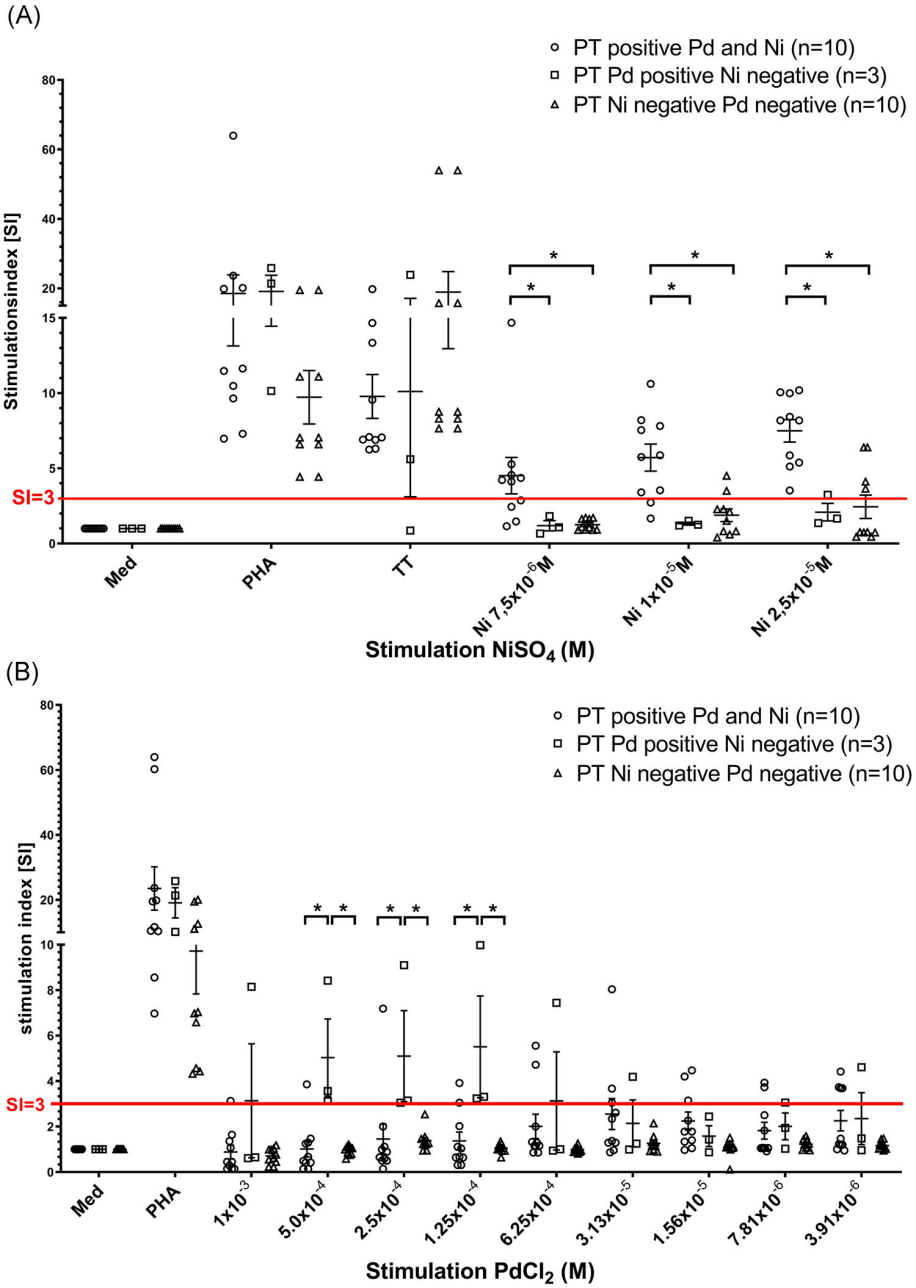
A, Proliferation of PBMC of patients with double‐positive PT to Ni and Pd (group 1), positive PT only to Pd (group 2), and negative PT to Ni and Pd (group 3) after 6 days of stimulation with Ni in three different concentrations. SI is given; a SI > 3 is regarded as positive reaction (red line); positive control PHA gave no differences in the three groups; *P < .05. B, Proliferation of PBMC of patients with double‐positive PT to Ni and Pd (group 1), positive PT only to Pd (group 2), and negative PT to Ni and Pd (group 3) after 6 days of stimulation with PdCl_2_ in nine different concentrations. SI is given; a SI > 3 is regarded as positive reaction (red line); positive control PHA gave no differences between the three groups; *P < .05. PBMC, peripheral blood mononuclear cells; PHA, phytohaemagglutinine; PT, patch test; SI, stimulation index

To optimize the LTT upon stimulation with Pd, 9 out of 20 different concentrations are seen in preceding control experiments as nontoxic and without “unspecific” stimulatory effect were used in the actual experiments. In the range of these nine different concentrations, we could detect proliferation rates of the patients PBMC. In accordance with Pichler and Tilch,^18^ as a positive result, we considered SI > 3 in at least one stimulation concentration. With regard to Ni, we could rely on three previously evaluated, optimal stimulation concentrations. Accordingly, all of the patients with a positive PT to Ni reacted in the LTT to Ni with a SI > 3 in at least one Ni concentration (Figure 1A), that is, all were considered positive. The sensitivity and specificity of the LTT test to Ni was up to 80% after stimulation with NiSO_4_ 1 × 10^−5^M. The data are shown in Table 4. The 3 only Pd PT positive patients and the 10 PT negative controls gave no significant LTT reaction to Ni. On the other hand, at higher Pd stimulation concentrations, the only Pd PT positive patients (group 2) had also reacted to Pd stimulation with a significant proliferation. Patients with concomitant Ni reaction in PT had however, remained negative in Pd stimulation (Figure 1B). The best results were obtained at a concentration range from 5 × 10^−4^M to 1.25 × 10^−4^M PdCl_2_. There was a perfect 100% sensitivity and specificity of the LTT test after stimulation with PdCl_2_ 2.5 × 10^−4^M. Interestingly patients with concomitant Ni PT reaction showed proliferation in lower Pd concentrations (below 3.13 × 10^−5^M; Table 4).

**Table 4:** Sensitivity and specificity of the LTT test to Ni and Pd calculated for the cut‐off level of the stimulation index, SI > 3

LTT		Ni and Pd PT positive vs PT negative			Single Pd PT positive vs PT negative		
Metal	Concentration	Sensitivity, %	Specificity, %	*P* value	Sensitivity, %	Specificity, %	*P* value
NiSO_4_	2.5 × 10^−5^M	100	60.00	.0108*	33.33	66.66	.999
NiSO_4_	1 × 10^−5^M	80.00	80.00	.0230*	0.00	80.00	.999
NiSO_4_	7.5 × 10^−6^M	60.00	100	.0108*	0.00	100	.999
PdCl_2_	1 × 10^−3^	33.33	100	.4737	33.33	100	.2308
PdCl_2_	5.0 × 10^−4^	10.00	100	.9999	66.67	100	.0385*
PdCl_2_	2.5 × 10^−4^	20.00	100	.4737	100	100	.0035**
PdCl_2_	1.25 × 10^−4^	40.00	100	.0867	33.33	100	.2308
PdCl_2_	6.25 × 10^−4^	40.00	100	.0867	33.33	100	.2308
PdCl_2_	3.13 × 10^−5^	50.00	100	.0325*	33.33	100	.2308
PdCl_2_	1.56 × 10^−5^	50.00	100	.0325*	00.00	100	.999
PdCl_2_	7.81 × 10^−6^	40.00	100	.0867	33.33	100	.2308
PdCl_2_	3.91 × 10^−6^	60.00	100	.0108**	33.33	100	.2308

Abbreviations: LTT, lymphocyte transformation test; PT, patch test.

*p < .05, **p < .01

### 3.3 Cytokine expression

**Figure 2:**
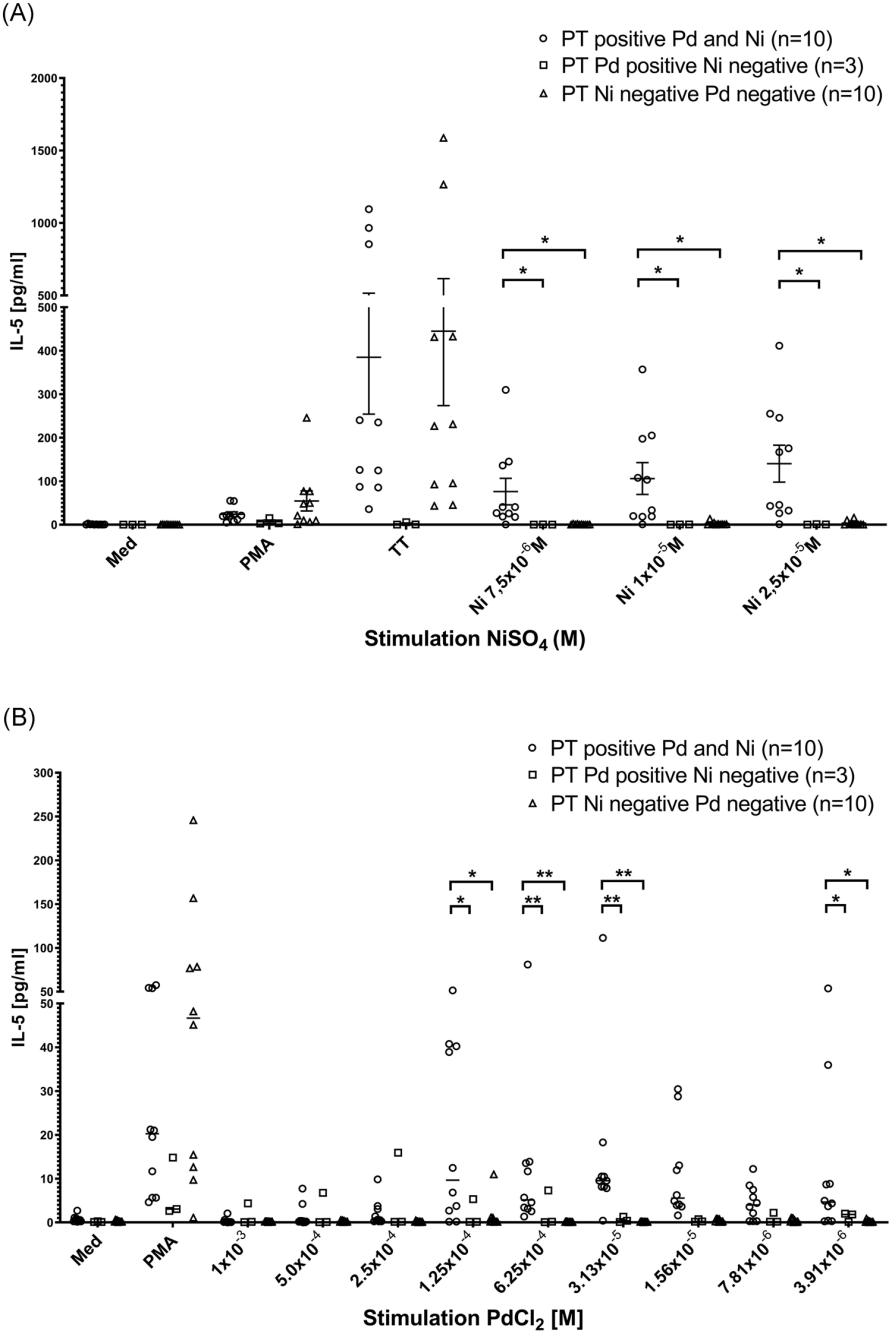
A, IL‐5 production of PBMC of patients with double‐positive PT to Ni and Pd (group 1), positive PT only to Pd (group 2) and negative PT to Ni and Pd (group 3) after 6 days of stimulation with NiSO_4_ in three different concentrations. IL‐5 concentration is given in pg/mL; positive control with PMA/ionomycion gave no differences between the three groups; *P < .05. B, IL‐5 production of PBMC of patients with positive PT both to Ni and Pd (group 1), positive PT only to Pd (group 2), and negative PT to Ni and Pd (group 3) after 6 days of stimulation with PdCl_2_ in nine different concentrations. IL‐5 concentration is given in pg/mL; positive control with PMA/ionomycion gave no differences between the three groups; *P < .05 and **P < .01. IL‐5, interleukin‐5; PBMC, peripheral blood mononuclear cells; PMA, phorbol‐myristate‐acetate; PT, patch test

There was no significant production of IL‐8, IFNγ, and IL‐17A after stimulation with Ni or Pd (data not shown). PBMC of patients who were PT positive both to Ni and Pd (group 1) produced IL‐5 in response to Ni and Pd stimulation (Figure 2A). IL‐5 production after Ni stimulation was more than 10‐fold increased in comparison to Pd stimulation. The sensitivity and specificity of the IL‐5 production after Ni stimulation was up to 90% and 100%, respectively (Table 5). Patients with concomitant Ni and Pd PT reactivity showed a lower but still significant IL‐5 production after Pd stimulation. Patients with single Pd contact allergy (group 2) and controls (group 3) did not show an IL‐5 response when using Pd stimulation (Table 5). The best discrimination level was reached by using a cut‐off level of 10 pg/mL IL‐5.

**Table 5:** Sensitivity and specificity of the IL‐5 production after stimulation with Ni and Pd calculated for the cut‐off level of 10 pg/mL IL‐5

IL‐5		Ni and Pd PT positive vs PT negative			Single Pd PT positive vs PT negative		
Metal	Concentration	Sensitivity, %	Specificity, %	*P* value	Sensitivity, %	Specificity, %	*P* value
NiSO_4_	2.5 × 10^−5^M	90.00	90.	.011*	0.00	90.00	.999
NiSO_4_	1 × 10^−5^M	90.00	100	.0001***	0.00	100	.999
NiSO_4_	7.5 × 10^−6^M	90.00	100	.0001***	0.00	100	.999
PdCl_2_	1 × 10^−3^	0.00	100	.999	0.00	100	.999
PdCl_2_	5.0 × 10^−4^	10.00	100	.999	33.33	100	.2308
PdCl_2_	2.5 × 10^−4^	0.00	100	.999	33.33	100	.2308
PdCl_2_	1.25 × 10^−4^	50.00	100	.0325*	0.00	100	.999
PdCl_2_	6.25 × 10^−4^	50.00	100	.0325*	0.00	100	.999
PdCl_2_	3.13 × 10^−5^	50.00	100	.0325*	0.00	100	.999
PdCl_2_	1.56 × 10^−5^	40.00	100	.0867	0.00	100	.999
PdCl_2_	7.81 × 10^−6^	10.00	100	.999	0.00	100	.999
PdCl_2_	3.91 × 10^−6^	20.00	100	.4737	0.00	100	.999

Abbreviations: IL‐5, interleukin‐5; PT, patch test.

*p < .05, ***p < .0001

## 4 DISCUSSION

In dentistry, Pd is increasingly used due to its good solubility with other metals, as well as its good mechanical properties.^33^ Due to its oral “biocompatibility,” in Japan, the government operates a specific mandate stating that all government‐subsidized dental alloys have to include a Pd content of at least 20%. Correspondingly this alloy is used in approximately 90% of all Japanese dental treatments.^34^

Apart from the oral cavity, Pd exposure may also occur following skin contact with consumer items.^15^ Pd is released from dental alloys; even teeth brushing may cause Pd release.^35^

Pd allergy is a potential elicitor of oral lesions associated with dental alloys. PT remains the gold standard for diagnosing oral disease resulting from an allergy to metallic dental materials. However, isolated Pd sensitization is rarely found, and concomitant PT reactivity to both Ni and Pd is reported.^10^ The review of Faurschou et al^15^ confirms that Pd allergy is common and nearly always observed together with Ni allergy. Analysis of the PT data as registered by the DKG in 2006‐2016 has shown that out of 1017 Pd PT positive individuals 782 (76.9%) had also reacted to Ni, that is, about 235 were monosensitized to Pd (Prof. Geier, personal communication, University of Göttingen).

Upon using a potentially more appropriate PT preparation, that is, sodium tetrachloropalladate^36^ in 23 Pd PT positive patients, cross‐reactivity with some of the nickel salts tested was found in 17 out of 23. Subsequently, Muris et al^6^ reported in 2014 that “significantly more Pd monosensitized patients were found in the oral disease group in the metal allergic contact dermatitis group.” The authors speculate, that cutaneous Ni exposure may preferentially induce Ni‐specific T cells that cross‐react with Pd as opposed to more often Pd‐specific reactivity by oral Pd exposure. This is also supported by two observations: (a) Ni allergy is more frequent in females, and our patients are almost only females. (b) Exposure to Pd from dental restorations is expected to be similar in men and women.^15^ With regard to Ni allergy, the LTT has been optimized for the evaluation of Ni reactivity in vitro.^19^, ^23^, ^24^ In addition, simultaneous assessment of released cytokines was suggested to better discriminate allergic from unspecific reactivity. Examples are IFNγ production or, more recently also, an assessment of TH2‐related cytokines. Jakobson et al^37^ found that PBMC from nickel‐allergic individuals responded to Ni with significantly increased production of IL‐4, IL‐5, IL‐13, and IFN‐γ, but not IL‐12, as compared with healthy controls. Minang et al^28^ demonstrated that IL‐10 is of importance for regulating the Ni‐mediated immune response to Ni. TH1 type (IFN‐γ) responses were supposed to be downregulated by IL‐10.^38^ Bordignon et al^39^ investigated blood samples from 40 patients who were patch tested. By enzyme‐linked immunospot (ELISpot) assay, they assessed IFN‐y and IL‐10 production in vitro and found that (a) PBMC of Ni and Pd PT negative patients only produced IL‐10 both to Ni and Pd stimulation, (b) PBMC of only Ni PT positive patients produced IFN‐y to Ni but only IL‐10 to Pd stimulation, (c) PBMC of Ni and Pd PT double‐positive patients produced IFN‐y to Ni but again only IL‐10 to Pd stimulation, (d) PBMC of only Pd PT positive patients produced IL‐10 to Ni and IFN‐y to Pd. They concluded that Pd is often “cross‐reacting” with dominant Ni sensitization and only isolated Pd PT positivity and distinctive cytokine pattern in vitro might indicate relevant Pd allergy. Unfortunately, in these experiments no proliferation assays were included.

In 2012 Muris et al^40^ again reported on Pd‐induced TH2 cytokine responses in vitro. Most of the patients in this study were however, double‐positive to Ni and Pd in PT.

Spiewak et al^30^ and Czarnobilska et al^41^ reported that Ni PT reactivity was reflected by IL‐5 production in vitro. Recently Summer et al^42^ could show that the determination of the IL‐5/IL‐8 response ratio in vitro could optimize detection of patients with Ni contact allergy. Thus, the aim of the present study was to compare PT and LTT reactivity with both Ni and Pd and to assess potential discrimination benefit from cytokine assessment. With the restriction of the rather low number of individuals tested, the use of Pd(II) chloride as reagent and the here not assessed IL‐13, we can draw the following conclusions:

As compared with Ni and Pd PT negative patients we could show that the 10 Pd and Ni double PT positive patients had clear‐cut LTT reactivity to Ni (10 out of 10) and none to Pd.

Based on Ni and Pd PT double‐negative patients we could demonstrate, that PBMC of these patients had neither LTT reactivity nor IL‐5 production both to Ni or to Pd stimulation. Furthermore, the PBMC of Pd PT positive Ni negative patients did not respond with IL‐5 production to Ni stimulation in vitro. As Pd stimulation in vitro had induced IL‐5 production in vitro to a much lower extent than Ni in the 10 Pd and Ni PT reactive patients, it seems that Ni sensitization is predominant and “isolated” relevant Pd PT reactivity—as opposed to Ni reactivity—is not characterized by IL‐5 response. Accordingly, Kobayashi et al^43^ reported from a mouse model on Pd‐sensitized animals, that after rechallenge (a) some of the isolated Pd‐specific clones produced Th1‐cytokines (like IFNy and TNF‐α), (b) IL‐10 production was also seen, but (c) TH2 cytokines (ie, IL‐4 and IL‐5) were not increased.

Taken together, marked IL‐5 response upon in vitro stimulation of PBMC by Ni is found in Ni allergic individuals. The LTT with additional analysis of the IL‐5 response can be a tool to distinguish between Pd and Ni cross‐reactivity and can help to identify patients with a Pd contact allergy, which means a proliferative response and at the same time lacking IL‐5 response after stimulation with Pd. This is different from Muris et al^6^ who also described 2 years after the above‐mentioned publication 13 Pd PT positive patients within the 16 IL‐5 reactors observed in a group of 71 patients. In a subsequent publication, Muris et al^44^ again reported on such IL‐5 reactivity, however, most of the patients were again PT double‐positive to Ni and Pd. Furthermore our here used LTT protocol is not comparable to the assays in their studies, which for example, also includes cytokine supplemented culture medium for the “cytokine experiments.”

In summary, the present study confirms, that Ni and Pd reactivity are often found simultaneously in PT, and that primary Ni sensitization is characterized by distinct LTT reactivity together with additional IL‐5 response in vitro. The combination of LTT test and IL‐5 measurement can be a helpful tool to distinguish between a “real” contact allergy to Pd or cross‐reactivity with Ni.

## CONFLICT OF INTERESTS

The authors declare that there are no conflict of interests.
